# Chemotherapy regimens induce inhibitory immune checkpoint protein expression on stem-like and senescent-like oesophageal adenocarcinoma cells

**DOI:** 10.1016/j.tranon.2021.101062

**Published:** 2021-03-22

**Authors:** Maria Davern, Noel E. Donlon, Andrew Sheppard, Fiona O’ Connell, Conall Hayes, Anshul Bhardwaj, Emma Foley, Dermot O’ Toole, Niamh Lynam-Lennon, Narayanasamy Ravi, John V. Reynolds, Stephen G. Maher, Joanne Lysaght

**Affiliations:** a Cancer Immunology and Immunotherapy Group, Department of Surgery, Trinity St. James's Cancer Institute, Trinity Translational Medicine Institute, St. James's Hospital campus, Dublin 8, Ireland; b Translational Gastrointestinal Research Group, Department of Surgery, Trinity St. James's Cancer Institute, Trinity Translational Medicine Institute, St. James's Hospital campus, Dublin 8, Ireland; c Department of Surgery, Trinity St. James's Cancer Institute, Trinity Translational Medicine Institute, St. James's Hospital, Trinity College Dublin, Dublin, Ireland; d Translational Radiobiology and Diagnostics Group, Department of Surgery, Trinity St. James's Cancer Institute, Trinity Translational Medicine Institute, St. James's Hospital campus, Dublin 8, Ireland; e Cancer Chemoradiation Group, Department of Surgery, Trinity St. James's Cancer Institute, Trinity Translational Medicine Institute, St. James's Hospital campus, Dublin 8, Ireland

**Keywords:** Oesophageal adenocarcinoma, Immune checkpoints, Chemotherapy, Stem-like, Senescence

## Abstract

Use of immune checkpoint inhibitors (ICIs) with chemotherapy to enhance responses in oesophageal adenocarcinoma (OAC) is an attractive approach. We identified subpopulations of OAC cells expressing inhibitory immune checkpoint (IC) ligands (PD-L1, PD-L2 and CD160) and receptors (PD-1, TIGIT, TIM-3, LAG-3 and A2aR) *in vitro* and in *ex vivo* biopsies. Combination chemotherapy regimens FLOT and CROSS promote a more immune-resistant phenotype through upregulation of IC ligands and receptors on OAC cells *in vitro*. Importantly, this study investigated if OAC cells, enriched for ICs exhibited a more stem-like and senescent-like phentoype. FLOT preferentially upregulates PD-L1 on a stem-like OAC cell phenotype, defined by ALDH activity*.* Expression of senescence-associated β-galactosidase is induced in a subpopulation of OAC cells following FLOT and CROSS chemotherapy treatment, along with enhanced expression of TIM-3 and A2aR ICs. Blockade of PD-1 signalling in OAC cells induced apoptosis and enhanced FLOT and CROSS chemotherapy toxicity *in vitro*. Upregulation of ICs on OAC cells following chemotherapy may represent potential mechanisms of chemo-immune resistance. Combination ICIs may be required to enhance the efficacy of chemotherapy and immunotherapy in OAC patients.

## Highlights

• OAC cells express several inhibitory immune checkpoint (IC) ligands and receptors.
• Chemotherapy upregulates IC ligands and receptors on the surface of OAC cells.
• ICs are enriched on stem-like and senescent OAC cells following chemotherapy.
• PD-1 blockade induced apoptosis and enhanced chemotherapy toxicity in OAC cells.

## Introduction

Oesophageal cancer (OC) is one of the deadliest cancers globally, ranked the third most common cancer of the gastrointestinal tract and sixth most commonly diagnosed cancer worldwide [1]. Oesophageal adenocarcinoma (OAC) affects the distal third of the oesophagus and is the predominant subtype of OC in Western countries and its incidence is rapidly increasing in this region [1].

Treatment options for OAC patients include the FLOT or MAGIC combination chemotherapy regimens before surgery (neoadjuvant) and after surgery (adjuvant) [2]. The FLOT chemotherapy regimen includes 3 different classes of drugs with distinct mechanisms of action, the anti-metabolite 5-fluorouracil (5-FU), a platinum-based DNA intercalator oxaliplatin and a taxane anti-microtubule docetaxel [2]. Leucovorin is a reduced form of folinic acid and is given as part of the FLOT regimen [3]. Leucovorin increases the toxicity of 5-FU by stabilising the binding of 5-FU to its drug target thymidylate synthetase prolonging the stability of 5-FU *in vivo* [4]. The MAGIC chemotherapy regimen includes a topoisomerase inhibitor epirubicin, a platinum-based DNA intercalator cisplatin and an anti-metabolite 5-FU (ECF) or capecitabine (a pro-drug of 5-FU) (ECX) [5]. Patients may also receive neoadjuvant chemotherapy with concurrent localised radiation, the CROSS regimen, which includes an anti-microtubule taxane paclitaxel and a platinum-based chemotherapy carboplatin in combination with daily 1.8 Gy doses of X-Ray radiation across 23 fractions with a cumulative dose of 41.4 Gy [6].

OAC patients that achieve a complete pathological response after receiving neoadjuvant chemotherapy or chemoradiation have a significantly increased overall survival [7,8]. However, ~70% of OAC patients don't achieve a complete pathological response and are subjected to treatment associated toxicities without any apparent therapeutic benefit [7,8]. This highlights the need to develop better treatment strategies to enhance the efficacy of these regimens.

Several studies have shown that immunogenic chemotherapies sensitise ‘cold’ tumours to immune checkpoint inhibitors (ICIs) by stimulating anti-tumour immune responses against the tumour [9]. The addition of ICIs to chemotherapy regimens prevents exhaustion of this chemotherapy-induced anti-tumour immune response [10]. Currently, clinical trials are ongoing in OAC testing whether combination immune checkpoint inhibitors (ICIs) and chemotherapy regimens will improve treatment efficacy. These trials include: NCT02730546 (neoadjuvant pembrolizumab (anti-programmed death-1 (anti-PD-1) + chemotherapy (carboplatin, 5-FU, oxaliplatin and paclitaxel) + irradiation), NCT02735239 (arm A: durvalumab (anti-PD-ligand 1 (anti-PD-L1) alone followed by chemotherapy (oxaliplatin + capecitabine), arm B: durvalumab + tremelimumab (anti-cytotoxic lymphocyte antigen-4 (anti-CTLA-4) + chemotherapy (oxaliplatin + capecitabine), arm C: combination durvalumab + chemotherapy (oxaliplatin + capecitabine), arm D: durvalumab + CROSS chemoradiation regimen or durvalumab or arm E: FLOT chemotherapy regimen), NCT02494583/KEYNOTE-062 (Pembrolizumab *versus* pembrolizumab + cisplatin + 5-FU *versus* placebo + cisplatin + 5-FU) and NCT02872116/CheckMate649 (nivolumab (anti-PD-1) + ipilimumab (anti-CTLA-4) *versus* nivolumab + oxaliplatin + 5-FU/capecitabine (FOLFOX/XELOX) *versus* FOLFOX/XELOX).

Interestingly, the central dogma that T cells and tumour cells exclusively express IC receptors and IC ligands respectively, no longer holds true [10]. Several studies have now shown that IC receptors are expressed on the surface of a subpopulation of cancer cells in a range of malignancies including OAC [11]. Emerging studies have shown that melanoma and breast cancer stem-like cells were enriched for the expression of PD-1 and PD-L1 [12], respectively. Recent studies have demonstrated that activation of both inhibitory IC ligands and receptors including PD-L1, PD-L2, TIM-3 and PD-1 on the surface of cancer cells promoted various immune-independent hallmarks of cancer such as an altered metabolism, proliferation, invasion and metastasis, DNA repair and chemoresistance [13], [14], [15], [16], [17], [18], [19], [20], and immune checkpoint blockade (ICB) suppressed various hallmarks of cancer including invasion, chemoresistance, proliferation, glycolysis and DNA repair [13], [14], [15], [16], [17], [18], [19], [20].

This study profiles the expression of a wide range of inhibitory IC ligands and receptors on the surface of OAC cells *in vitro* and in *ex vivo* biopsies to identify potential therapeutic targets. The effect of clinically relevant combination chemotherapies (FLOT, CROSS chemotherapy (CROSS CT) and MAGIC) on the expression of ICs on OAC was assessed *in vitro*. Additionally, we also investigated the phenotype of OAC cells expressing ICs, specifically the stem-like and senescent-like properties of OAC cells expressing IC ligands and receptors. In light of the emerging studies highlighting novel immune-independent functions for ICs in cancer cells, we also investigated if blockade of PD-1 signalling in OAC cells reduces the viability of OAC cells and enhances chemotherapy toxicity *in vitro*.

## Materials and methods

### Acquisition of human OAC tumour tissue biopsies

All patients involved in this study were enrolled from 2018–2020. Treatment-naïve tumour tissue biopsies were obtained from OAC patients undergoing endoscopy at St. James's Hospital (*n* = 10) at time of diagnosis prior to initiation of chemotherapy or radiotherapy. Post-FLOT chemotherapy-treated (*n* = 5) and post-CROSS chemoradiotherapy-treated OAC (*n* = 5) tumour tissue biopsies were obtained at time of surgical tumour resection. The group consisted of 11 males and 5 females, with an average age of 63.6 years. The patient demographics are detailed in Table S1. The work was performed in accordance with the Code of Ethics of the World Medical Association (Declaration of Helsinki) for experiments involving human samples. Patients provided informed consent for sample and data acquisition, and the study received full ethical approval from the St. James's Hospital/AMNCH Ethical Review Board. Patient samples were pseudonymised to protect the privacy rights of the patients.

### OAC tumour tissue digestion

Biopsies were enzymatically digested to perform OAC cell phenotyping. Briefly, tissue was minced using a scalpel and digested in collagenase solution (2 mg/ml of collagenase type IV (Sigma) in Hanks Balanced Salt Solution (GE healthcare) supplemented with 4% (v/v) foetal bovine serum) at 37 °C and 1500 rpm on an orbital shaker. Tissue was filtered and washed with FACs buffer (PBS containing 1% foetal bovine serum and 0.01% sodium azide). Cells were then stained for flow cytometry.

### Cell culture of OAC cell lines

Human Het1A cells, OE33 cells and SK-GT-4 cells were purchased from European Collection of Cell Cultures. Het1A cells were grown in flasks precoated with a mixture of fibronectin (0.01 mg/ml, Merck, Germany), collagen type I (0.03 mg/ml, Corning, USA) and bovine serum albumin (0.01 mg/ml, Sigma, USA) dissolved in PBS and cultured in serum free Bronchia/Trachea Epithelial Cell Growth Medium (511–500, Merck, Germany). Het1A cells were detached from flask using accutase (Sigma, USA) diluted 1:15 in PBS. OE33 and SK-GT-4 cells were grown in RPMI 1640 medium with 2 mM L-glutamine (Gibco) and supplemented with 1% (v/v) penicillin-streptomycin (50 U/ml penicillin 100 μg/ml streptomycin) and 10% (v/v) foetal bovine serum (Gibco). All cell lines were maintained in a humidified chamber at 37 °C 5% CO_2_ and were tested regularly to ensure mycoplasma negativity.

### Cell viability CCK-8 assay

A CCK-8 assay (Sigma, USA) was used to assess the effect of single agent 5-FU, oxaliplatin, docetaxel, epirubicin, cisplatin, carboplatin and paclitaxel on the viability of OE33 and SK-GT-4 cells and combination chemotherapy regimens FLOT, CROSS CT and MAGIC. Additionally, the effect of nivolumab, atezolizumab or dual nivolumab-atezolizumab in the absence or presence of FLOT and CROSS CT regimens on the viability of OE33 and SK-GT-4 cells was investigated. 5 × 10^3^ OAC cells were adhered in a 96 well plate at 37 °C, 5% CO2 overnight. Cells were treated with increasing doses of either single agent chemotherapy or a combination of chemotherapies including FLOT (5-FU, oxaliplatin, docetaxel), CROSS CT (carboplatin and paclitaxel) or MAGIC (epirubicin, cisplatin and 5-FU) at a range of inhibitory concentrations (IC_10_, IC_25_, IC_50_) (Table S2 and Table S3) for 48 h. The doubling time of OE33 and SK-GT-4 cells is 33 and 36 h, respectively, therefore, a 48 h time point allowed sufficient time for the chemotherapies to be incorporated into the cells and induce cytotoxicity. Additionally, cells were treated with and without FLOT or CROSS CT regimen in the absence or presence of single agent atezolizumab (10 ug/ml), nivolumab (10 ug/ml) or dual atezolizumab (10 ug/ml) and nivolumab (10 ug/ml). An IC_25_ dose of each chemotherapy in the FLOT and CROSS CT regimen was used to treat OE33 cells. An IC_50_ and IC_25_ dose of each chemotherapy in the FLOT and CROSS CT regimens respectively, were used to treat the SK-GT-4 cells as shown in Table S3. 5  μl of CCK-8 solution was added to each well, followed by a 1.5 h incubation in the dark at 37 °C, 5% CO2. The optical density at 450 nm and 650 nm (reference wavelength) was measured using the Versa Max microplate reader (Molecular Devices, Sunnyvale, CA, USA) to determine a viable cell number. All of the data were analysed from three independent experiments.

### Annexin V and propidium iodide assay

Apoptosis was assessed using annexin V-FITC and propidium iodide (PI) staining by flow cytometry. OE33 cells and SK-GT-4 cells were treated with nivolumab (10 ug/ml), atezolizumab (10 ug/ml) or dual nivolumab (10 ug/ml) and atezolizumab (10 ug/ml) or untreated in RPMI medium control for 48 h. OE33 cells and SK-GT-4 cells were treated with and without FLOT or CROSS CT regimen in the absence or presence of single agent nivolumab (10 ug/ml), atezolizumab (10 ug/ml) or dual nivolumab (10 ug/ml) and atezolizumab (10 ug/ml) for 48 h. Cells were stained with Annexin V-FITC (Biolegend, USA) and 1:4000 dilution of PI (Invitrogen, Carlsbad, CA, USA), and samples were acquired using BD FACs CANTO II (BD Biosciences) using Diva software and analysed using FlowJo v10 software (TreeStar, Inc., Ashland, Oregon).

### Flow cytometry staining for *in vitro* cell lines and *ex vivo* OAC biopsies

Het1A cells were detached using accutase (Sigma, USA) diluted 1:15 in PBS and OE33 and SK-GT-4 cells were trypsinised and stained with zombie aqua viability dye (Biolegend, USA) for gating on live cells. Antibodies used for cell lines included: PD-L1-FITC, PD-L2-PE and CD54-PE/Cy5.5 (BD Biosciences, USA), TIM-3-biobrightFITC (Miltenyi, USA), CD160-PERCPCy5.5, TIGIT-PE/Cy7, LAG-3-FITC, PD-1-PE/Cy7 (Biolegend, USA), A2aR-PE and β-galactosidase-AF405 (Bio-techne, USA). OE33 and SK-GT-4 cells were fixed with 1% paraformaldehyde solution and acquired using BD FACs CANTO II (BD Biosciences) using Diva software and analysed using FlowJo v10 software (TreeStar Inc.).

OAC tumour tissue biopsies were stained with zombie aqua viability dye (Biolegend, USA) and the following cell surface antibodies: PD-L1-FITC and TIM-3-AF647 (BD Biosciences, USA), CD45-APC/Cy7, CD31-AF700, PD-L2-BV421, CD160-PERCPCy5.5, TIGIT-BV605, LAG-3-BV650 and PD-1-PE/Cy7 (Biolegend, USA) and A2aR-PE (Bio-techne, USA). Cells were fixed with 1% paraformaldehyde solution, washed with FACs buffer, resuspended in FACs buffer and acquired using BD LSR Fortessa flow cytometer (BD Biosciences) using Diva software and analysed using FlowJo v10 software (TreeStar Inc.). OAC cells were identified as CD31^−^CD45^−^ cells excluding endothelial cells and immune cells, respectively as described previously [21]. Gating strategy used for analysis is shown in Fig. S1.

### Aldehyde dehydrogenase (ALDH) assay

Aldehyde dehydrogenase (ALDH) enzyme activity was assessed using the Aldefluor® assay (Stem Cell Technologies), according to the manufacturer's instructions. Briefly, cells were trypsinised and resuspended at a density of 1 × 10^6^ cells/mL in Aldefluor® assay buffer containing ALDH substrate (bodipy-aminoacetaldehyde) (5 μL/mL). Immediately following this, half of the resuspended cells were added to a tube containing the ALDH inhibitor diethylaminobenzaldehyde (DEAB) to provide a negative control. Cells were acquired using BD FACs CANTO II (BD Biosciences) using Diva software and analysed using FlowJo v10 software (TreeStar Inc.).

### Statistical analysis

Data were analysed using GraphPad Prism (GraphPad Prism, San Diego, CA, USA) software and was expressed as mean ± SEM. Statistical differences between two treatments in a particular cell line were analysed using a paired parametric Student's *t*-test. To compare the statistical differences between two different cell lines an unpaired parametric *t*-test was conducted. To compare differences between patients and different treatments an unpaired non-parametric *t*-test was conducted. Statistical significance was determined as *p* ≤ 0.05. To determine if IC expression on OAC cells in the treatment-naïve setting and post-treatment setting correlated with patient clinical features, Spearman correlations were performed to analyse correlation data.

## Results

### A subpopulation of normal oesophageal epithelial cells and OAC cells express inhibitory IC ligands and inhibitory IC receptors *in vitro*

Emerging studies have shown that cancer cells not only express inhibitory IC ligands but also inhibitory IC receptors including PD-1, TIGIT and TIM-3 in OAC [11], melanoma [22], pancreatic [15], gastric [18,20] cervical [23], lung [16,24], ovarian [25], endometrial [25] and colorectal cancer [26]. In addition, IC receptor TIM-3 has also been identified on normal gastric epithelial cells as well as normal cervical epithelial cells. Therefore, we screened normal oesophageal epithelial (Het1A cells) and OAC cells for the expression of both inhibitory IC ligands and receptors. OE33 and SK-GT-4 OAC cell lines used in this study were established from tumours that had progressed from the pre-malignant Barrett's oesophagus condition to OAC. While the OE33 cells are poorly differentiated, the SK-GT-4 cells are well differentiated, which helps to encapsulate the heterogeneous nature of OAC.

**Fig. 1:**
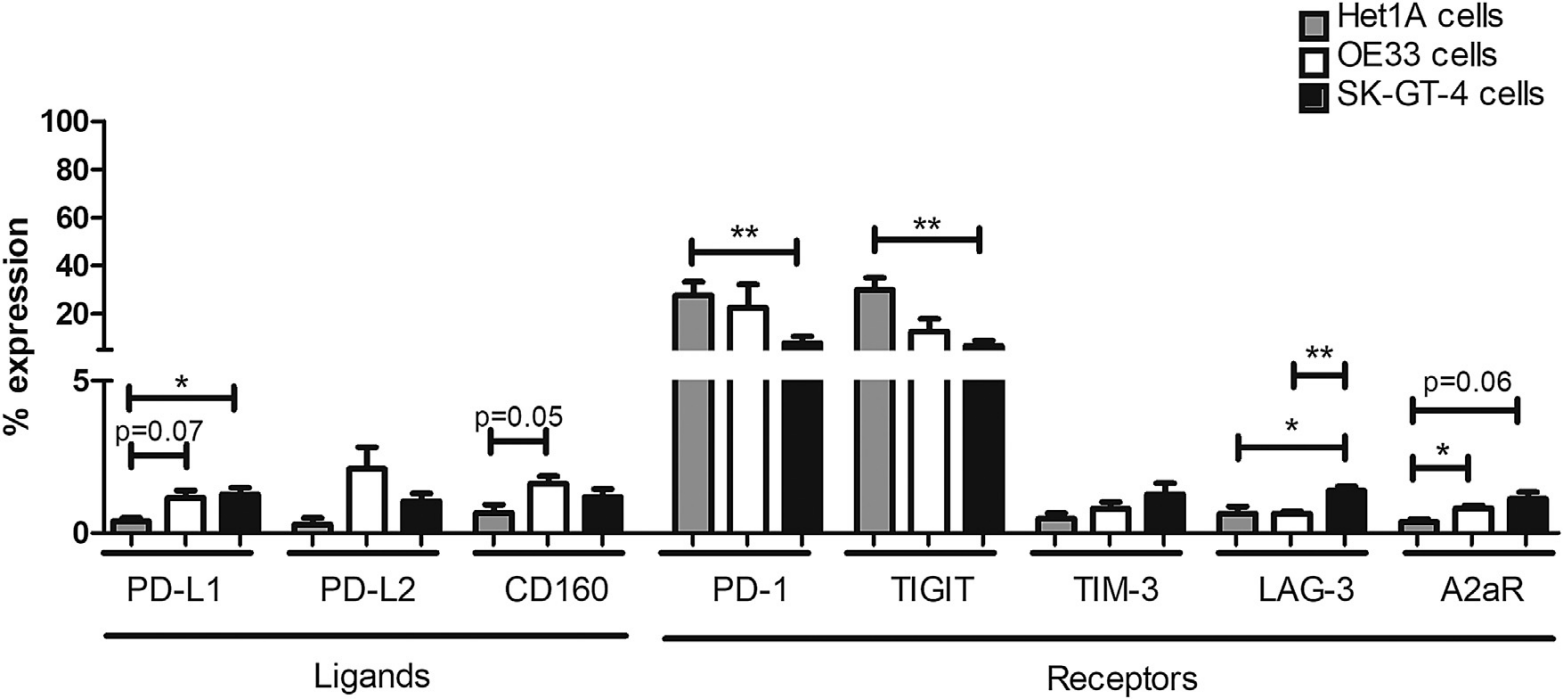
Normal oesophageal epithelial cells and OAC cells basally express both inhibitory IC ligands and receptors *in vitro*. Het1A cells, OE33 cells and SK-GT-4 cells were screened for the percentage expression of inhibitory IC ligands PD-L1, PD-L2, CD160 (*n* = 3) and inhibitory IC receptors PD-1, TIGIT, TIM-3, LAG-3 and A2aR (*n* = 3) *in vitro* by flow cytometry. Mann Whitney test *p < 0.05 and **p < 0.01.

Het1A cells and both OAC cell lines expressed low levels of PD-L1 (Het1A 0.38 ± 0.1%, OE33 1.16 ± 0.2% and SK-GT-4 cells 1.29 ± 0.2%), PD-L2 (Het1A 0.29 ± 0.2%, OE33 2.11 ± 0.7% and SK-GT-4 cells 1.06 ± 0.2%) and CD160 (Het1A 0.67 ± 0.3%, OE33 1.63 ± 0.2% and SK-GT-4 cells 1.18 ± 0.3%) (Fig. 1.). PD-1 is also expressed by Het1A cells and both OAC cell lines (Het1A 27.80 ± 5.6%, OE33 22.64 ± 9.6% and SK-GT-4 cells 7.88 ± 2.8%), as are TIGIT (Het1A 30.00 ± 5.1%, OE33 12.76 ± 5.2% and SK-GT-4 cells 6.77 ± 2.1%), TIM-3 (Het1A 0.48 ± 0.2%, OE33 0.80 ± 0.2% and SK-GT-4 cells 1.28 ± 0.4%), LAG-3 (Het1A 0.64 ± 0.2%, OE33 0.6500 ± 0.1% and SK-GT-4 0.64 ± 0.2%) and A2aR (Het1A 0.37 ± 0.1%, OE33 0.82 ± 0.1% and SK-GT-4 cells 1.13 ± 0.2%) (Fig. 1.). Furthermore, the expression profile of ICs was significantly different between the Het1A cells and the two OAC cell lines. A significantly higher percentage of SK-GT-4 cells expressed PD-L1 and LAG-3 compared with Het1A cells (Fig. 1.). In addition, there was a significantly higher percentage of OE33 cells positive for CD160 and A2aR compared with Het1A cells (Fig. 1.). Interestingly, PD-1 and TIGIT were expressed on a significantly higher percentage of Het1A cells compared with the SK-GT-4 cell line (Fig. 1.). A higher percentage of SK-GT-4 cells expressed LAG-3 compared with the OE33 cells (Fig. 1.). There were no other significant differences in IC expression levels for the other inhibitory IC proteins between the cell lines.

### Chemotherapy promotes a more immune-resistant phenotype through upregulation of inhibitory IC ligands on OAC cells *in vitro*

**Fig. 2:**
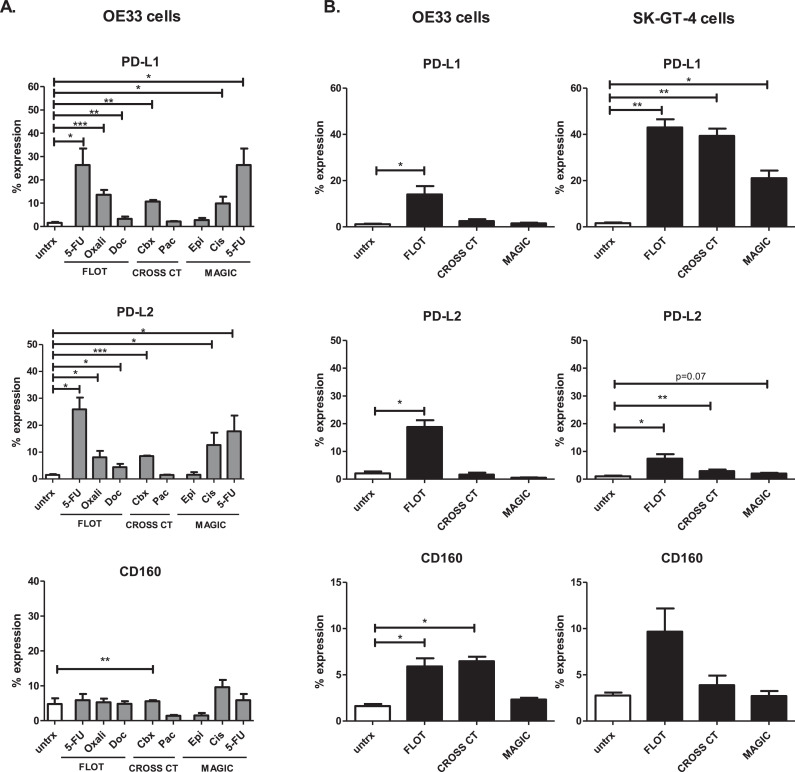
Chemotherapy regimens significantly alter the surface expression of a range of inhibitory IC ligands on the surface of viable OAC cells. OE33 cells and SK-GT-4 cells were untreated (untrx) or treated with clinically-relevant single agent chemotherapies (A) or combination chemotherapy regimens FLOT, CROSS CT and MAGIC (B) for 48 h *in vitro* and the percentage of viable OE33 and SK-GT-4 cells expressing inhibitory IC ligands was determined by flow cytometry. Single agent chemotherapies included 5-FU, oxaliplatin (oxali) and docetaxel (doc) (FLOT regimen), carboplatin (cbx) and paclitaxel (pac) (CROSS CT regimen) and epirubicin (epi), cisplatin (cis) and 5-FU (MAGIC regimen). Paired parametric *t*-test, (*n* = 3) *p < 0.05, **p < 0.01 and ***p < 0.001.

Previous studies have shown that single agent cisplatin and 5-FU increase PD-L1 on the surface of lung cancer [27] and OAC cells [28] *in vitro* respectively, which may drive immune evasion and resistance to chemotherapy. Therefore, we sought to investigate the effect of clinically-relevant single agent chemotherapies on the expression of a range of inhibitory IC ligands (PD-L1, PD-L2 and CD160) on the surface of OE33 cells by flow cytometry. Importantly, we also assessed the effect of the combination chemotherapy regimens FLOT, CROSS CT and MAGIC on the expression of a range of inhibitory IC ligands on the surface of OE33 and SK-GT-4 cells by flow cytometry. For single agent chemotherapies an IC_50_ dose was used. To obtain IC_50_ doses for combination FLOT, CROSS CT and MAGIC regimens; OE33 and SK-GT-4 cells were treated with increasing doses of single agent chemotherapies that comprise the FLOT, CROSS CT and MAGIC regimens to identify IC_10_, IC_25_ and IC_50_ doses for each chemotherapy at a 48 h time point (Fig. S2.). Subsequently, OE33 and SK-GT-4 cells were then treated with the combination regimens for 48 h using an IC_10_, IC_25_ or IC_50_ dose of each drug to obtain an inhibitory concentration of combination doses which killed 50% of the cells (Fig. S3.). These combination doses that reduced the viability of OE33 and SK-GT-4 cells by 50% were used for all the experiments in this study. Single agent 5-FU, oxaliplatin and docetaxel significantly increased the percentage of OE33 cells expressing PD-L1 (untreated 1.31 ± 0.5%, 5-FU 26.35 ± 6.4% (p=0.01), oxaliplatin: 13.59 ± 2.1% (p=0.0007) and docetaxel: 3.40 ± 2.1% (p=0.02)), (Fig. 2A.). The combination FLOT regimen also significantly increased PD-L1 expression on OE33 cells (1.16 ± 0.2% vs. 14.08 ± 3.5%, (p = 0.01)) and SK-GT-4 cells (1.62 ± 0.3% vs. 43.0 ± 3.6%, (*p* = 0.01)), (Fig. 2B.). Similarly, single agent 5-FU, oxaliplatin and docetaxel as well as the combined FLOT regimen significantly increased the percentage of OE33 and SK-GT-4 cells expressing PD-L2 (Fig. 2A. and Fig. 2B.). Single agent carboplatin (but not paclitaxel or CROSS CT) significantly increased PD-L1 and PD-L2 expression on the surface of OE33 cells. However, CROSS CT significantly increased PD-L1 and PD-L2 on the surface of SK-GT-4 cells (Fig. 2A.). Only single agent carboplatin significantly increased CD160 on the surface of OE33 cells, as did FLOT and CROSS CT regimens. There was no significant changes observed for CD160 expression on the SK-GT-4 cell line (Fig. 2A.). Single agent cisplatin and 5-FU but not epirubicin significantly increased PD-L1 expression on OE33 cells (untreated 1.44 ± 0.6% vs. cisplatin 12.65 ± 4.6% (p=0.04) and 5-FU 26.35 ± 6.4% (*p* = 0.01)), (Fig. 2A.). Similar results were observed for PD-L2 on OE33 cells (Fig. 2A.). The combination MAGIC regimen had no effect on IC ligand expression on OE33 cells, however PD-L1 was significantly increased post-MAGIC treatment on the surface of SK-GT-4 cells (untreated 1.62 ± 0.3% vs. MAGIC 21.13 ± 3.3%, (*p* = 0.03)), (Fig. 2B.).

Overall, 5-FU and the combination FLOT regimen had the greatest effect at upregulating inhibitory IC ligands, in particular PD-L1, on the surface of OAC cells *in vitro*.

### Chemotherapy promotes a more immune-resistant phenotype through upregulation of inhibitory IC receptors on OAC cells *in vitro*

**Fig. 3:**
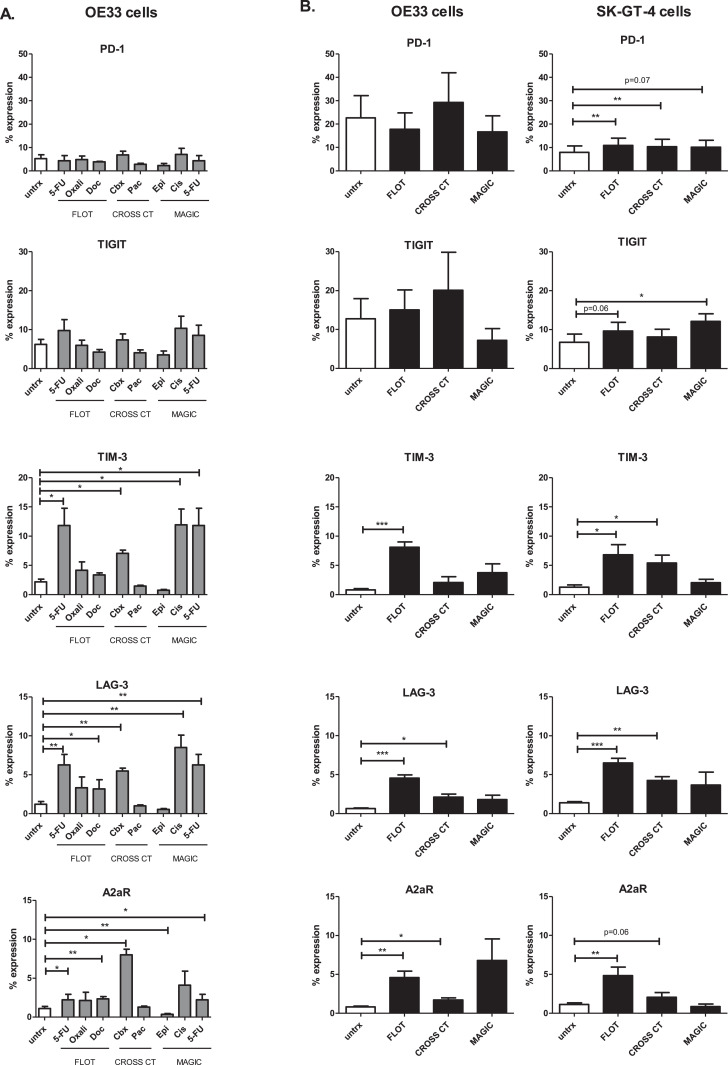
Chemotherapy regimens significantly increase a range of inhibitory IC receptors on the surface of viable OAC cells. OE33 cells and SK-GT-4 cells were untreated (untrx) or treated with clinically-relevant single agent chemotherapies (A) or combination chemotherapy regimens FLOT, CROSS CT and MAGIC (B) for 48 h *in vitro* and the percentage of viable OE33 and SK-GT-4 cells expressing inhibitory IC receptors was determined by flow cytometry (*n* = 4). Single agent chemotherapies included 5-FU, oxaliplatin (oxali) and docetaxel (doc) (FLOT regimen), carboplatin (cbx) and paclitaxel (pac) (CROSS CT regimen) and epirubicin (epi), cisplatin (cis) and 5-FU MAGIC regimen). Paired *t*-test, *p < 0.05, **p < 0.01 and ***p < 0.001.

As we demonstrated that single agent chemotherapy and combination chemotherapy regimens significantly altered the expression profile of inhibitory IC ligands on the surface of OAC cells, we also assessed the effect of these chemotherapies on the expression profile of a panel of inhibitory IC receptors on OAC cells. Treatment with single agent chemotherapies or combination chemotherapy regimens did not significantly affect the percentage of OE33 cells expressing PD-1 or TIGIT (Fig. 3A.). However, FLOT and CROSS CT regimens significantly increased PD-1 expression on SK-GT-4 cells (untreated: 7.88 ± 2.7% vs. FLOT: 10.85 ± 3.2%, (p=0.002) and CROSS CT: 10.30 ± 3.2%, (p=0.01)), and MAGIC significantly increased the expression of TIGIT on the surface of SK-GT-4 cells (untreated: 6.77 ± 2.1% vs. MAGIC: 12.11 ± 1.9%, (p=0.02)), (Fig. 3A.). Single agent 5-FU (but not oxaliplatin or docetaxel) and the combined FLOT regimen significantly increased the percentage of OE33 cells expressing TIM-3 (untreated: 2.6 ± 0.6% vs. 5-FU: 11.81 ± 2.9%, (p=0.04)), and untreated: 0.80 ± 0.2% vs. FLOT: 8.10 ± 0.9% (*p* = 0.0003)) (Fig. 3A. and Fig. 3B.). Similarly, FLOT significantly increased the percentage of SK-GT-4 cells expressing TIM-3 (Fig. 3A. and 3B). Single agent carboplatin (but not paclitaxel or CROSS CT) significantly increased the percentage of OE33 cells expressing TIM-3 (Fig. 3A. and 3B). Similarly, CROSS CT significantly increased the percentage of SK-GT-4 cells expressing TIM-3 (Fig. 3A. and 3B). The MAGIC regimen did not significantly affect the percentage of OE33 or SK-GT-4 cells expressing TIM-3, however, single agent cisplatin and 5-FU (but not epirubicin) significantly increased the percentage of OE33 cells expressing TIM-3 (Fig. 3A. and 3B). The percentage of OE33 cells expressing LAG-3 was significantly increased following single agent 5-FU and docetaxel treatment (but not oxaliplatin) as well as combination FLOT treatment (Fig. 3A. and Fig. 3B.). Furthermore, carboplatin (but not paclitaxel) significantly increased LAG-3 expression in OE33 cells, as did the CROSS CT regimen. Additionally, FLOT and CROSS significantly increased LAG-3 expression in the SK-GT-4 cells (Fig. 3A. and 3B). MAGIC had no effect on the percentage of OE33 and SK-GT-4 cells expressing LAG-3, however, single agent cisplatin and 5-FU (but not epirubicin) significantly increased LAG-3 expression in OE33 cells (Fig. 3A. and 3B). 5-FU and docetaxel (but not oxaliplatin) significantly increased the percentage of OE33 cells expressing A2aR, as did the combined FLOT regimen. Furthermore, single agent carboplatin (but not paclitaxel) significantly increased the percentage of OE33 cells expressing A2aR, as the CROSS CT regimen (Fig. 3A. and 3B). While epirubicin significantly decreased the percentage of OE33 cells expressing A2aR and 5-FU significantly increased the expression of A2aR on OE33 cells, (single agent cisplatin or the combination MAGIC regimen had no significant effect on A2aR expression). Only the FLOT regimen significantly increased the percentage of SK-GT-4 cells expressing A2aR (Fig. 3A. and 3B).

Similarly, in addition to single agent 5-FU and combination FLOT chemotherapy regimen having the greatest effect in upregulating IC ligand expression on OAC cells, 5-FU and FLOT also had the greatest effect in upregulating inhibitory IC receptors in particular LAG-3, TIM-3 and A2aR on the surface of OAC cells *in vitro*.

### The FLOT chemotherapy regimen enhances a stem-like phenotype and preferentailly upregulates PD-L1 and TIM-3 on the surface of stem-like OAC cells *in vitro*

**Fig. 4:**
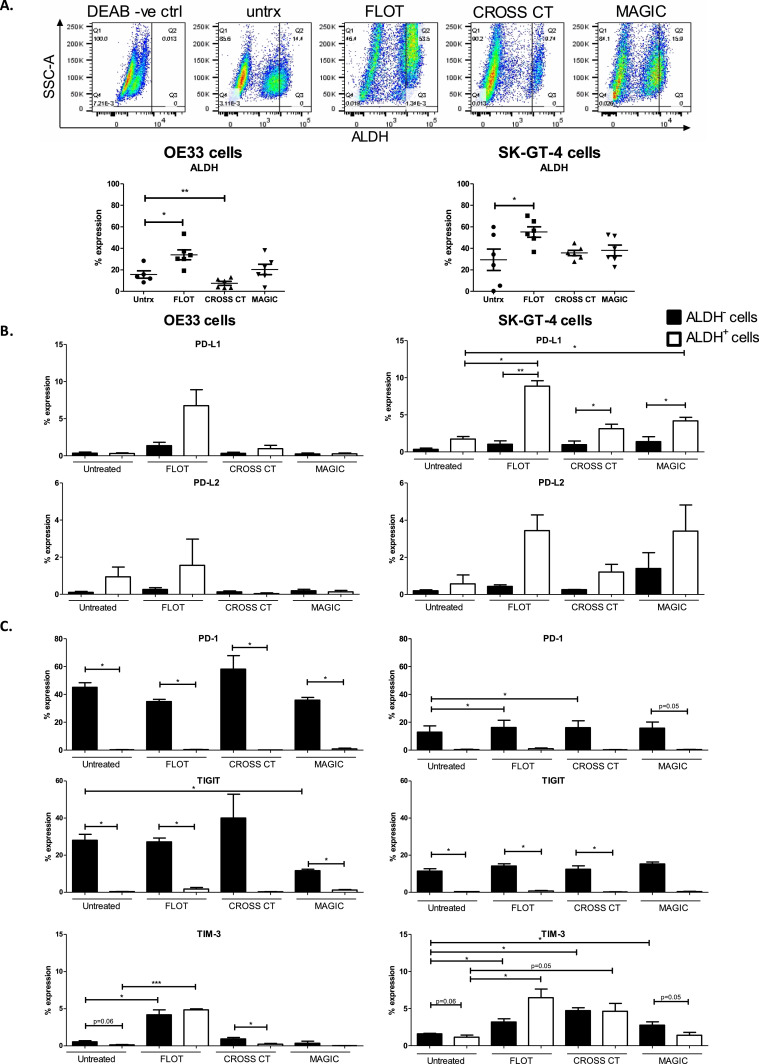
The FLOT chemotherapy regimen significantly increases ALDH activity and preferentially upregulates PD-L1 and TIM-3 on the surface of stem-like OAC cells *in vitro*. OE33 cells and SK-GT-4 cells were untreated or treated with FLOT, CROSS CT and MAGIC chemotherapy regimens for 48 h *in vitro.* Representative dot plots showing the level of ALDH activity in ALDH negative DEAB control (DEAB -ve ctrl), basally (untrx) and following FLOT, CROSS CT and MAGIC chemotherapy treatment in OE33 cells (A). The level of ALDH activity was determined in viable OAC cells by flow cytometry (*n* = 6) (A). The percentage of viable ALDH^−^ non-stem-like and ALDH^+^ stem-like OE33 and SK-GT-4 cells expressing inhibitory IC ligands (B) and inhibitory IC receptors (C) was determined by flow cytometry (*n* = 3). Paired parametric *t*-test, *p < 0.05, **p < 0.01 and ***p < 0.001.

Our data suggests that chemotherapy regimens enhance a more immune resistant phenotype in OAC cells, demonstrated by the upregulation of inhibitory IC ligands and receptors on OAC cells following FLOT and CROSS CT treatment. Therefore, we sought to investigate if chemotherapy regimens could enhance a more stem-like phentoype in OAC cells, which are thought to be responsible for tumour recurrence and treatment resistance [29,30]. OE33 and SK-GT-4 cells were treated with FLOT, CROSS CT and MAGIC regimens for 48 h and the level of ALDH activity was measured, which is an established marker of stemness in OAC [31] (Fig. 4A.). Following FLOT treatment, ALDH activity in OE33 cells was significantly increased (untreated: 15.59 ± 3.4% vs. FLOT: 33.97 ± 4.6%, (p=0.01) (Fig. 4A.). However, following CROSS CT the level of ALDH activity was significantly decreased in OE33 cells (untreated: 15.59 ± 3.4% vs. 7.42 ± 1.8%, (p=0.02)) (Fig. 4A.). MAGIC did not significantly alter ALDH activity in OE33 cells (Fig. 4A.). Following FLOT treatment of SK-GT-4 cells, ALDH activity was significantly increased (untreated: 29.40 ± 9.9% vs. FLOT: 55.17 ± 4.9%, (*p* = 0.02)) (Fig. 4A.). However, CROSS CT and MAGIC did not significantly alter ALDH activity in SK-GT-4 cells (Fig. 4A.).

Given that chemotherapy regimens upregulated inhibitory IC ligands and receptors on a subpopulation of OAC cells and that FLOT chemotherapy regimen enhanced a more stem-like phenotype in OAC cells, we examined if OAC cells exhibiting a stem-like phenotype had an altered expression of inhibitory IC ligands and receptors basally and following chemotherapy treatment. Figure S4. details the gating strategy for assessing the expression of IC ligands and receptors on the surface of ALDH^−^ and ALDH^+^ OAC cells *in vitro*. We found that inhibitory IC ligands and receptors are expressed on stem-like and non-stem-like OAC cells basally and following chemotherapy treatment (Fig. 4B). Basally, there was no significant difference in the percentage of OE33 or SK-GT-4 cells expressing PD-L1 and PD-L2 between the stem-like and non-stem-like compartment in OAC cells (Fig. 4B.). Following FLOT chemotherapy treatment, PD-L1 was significantly increased on the surface of ALDH^+^ stem-like SK-GT-4 cells (untreated ALDH^+^: 1.73 ± 0.4% vs. FLOT ALDH^+^: 8.86 ± 0.7%, (*p* = 0.01)), (Fig. 4B.). Basally, there was a significantly higher percentage of ALDH^−^ non-stem-like OE33 cells expressing PD-1, TIGIT and TIM-3 compared to the ALDH^+^ stem-like OE33 cells (PD-1: 45.07 ± 3.3% vs. 0.25 ± 0.1%, respectively (*p =* 0.005), TIGIT: 28.00 ± 3.2% vs. 0.32 ± 0.1%, respectively (*p =* 0.01) and TIM-3: 0.55 ± 0.1% vs. 0.13 ± 0.04%, respectively (*p =* 0.05)) (Fig. 4C.). Similarly, there was a significantly higher percentage of ALDH^−^ non-stem-like SK-GT-4 cells expressing TIGIT compared to the ALDH^+^ stem-like SK-GT-4 cells (11.42 ± 1.3% vs. 0.34 ± 0.1%, respectively (*p* = 0.01)) (Fig. 4C.). Following FLOT, CROSS CT and MAGIC chemotherapy treatment the percentage of OE33 cells expressing PD-1 and TIGIT was significantly higher in the ALDH^−^ non-stem-like compartment compared to the ALDH^+^ stem-like compartment basally (Fig. 4C.). Similar findings were observed for TIGIT in the SK-GT-4 cell line (Fig. 4C.). The percentage of cells expressing PD-1 was significantly increased in the ALDH^−^non-stem-like compartment of SK-GT-4 cells following FLOT and CROSS CT treatment (untreated ALDH^−^: 12.91 ± 4.5%, FLOT ALDH^−^: 16.25 ± 5.2% (p=0.03) and CROSS CT ALDH^−^: 16.19 ± 4.9% (p=0.01)). TIM-3 was significantly increased on ALDH^+^ stem-like SK-GT-4 cells following FLOT (untreated ALDH^+^: 1.15 ± 0.3% and FLOT ALDH^+^: 6.47 ± 1.2%, (*p* = 0.03)) (Fig. 4C.).

Overall, the FLOT regimen enhanced a stem-like phenotype in both OE33 and SK-GT-4 cells demonstrated by an increase in ALDH activity. Inhibitory ICs PD-L1 and TIM-3 were enriched in the stem-like compartment of OAC cells following chemotherapy treatment. However, PD-1 and TIGIT were preferentialy expressed on non-stem-like OAC cells basally and following chemotherapy treatment.

### The FLOT chemotherapy regimen enhances a senescent-like phenotype and upregulates A2aR on the surface of senescent-like OAC cells *in vitro*

Senescent cancer cells play an important role in conferring treatment resistance and in orchestrating a tumour-promoting milieu of immuosuppressive cells within the tumour microenvirnoment via the secretion of a range of pro-inflammatory markers known as the senescence-associated secretory phentoype (SASP). Characterisation of senescent cells can be performed by assessing multiple markers such as an enlarged morphology, activation of p53-p21 and/or p16-Rb tumour suppressor pathways, induction of p16^INK4a^, the presence of persistent DNA damage response, an increase in β-galactosidae (β-gal^+^) expression, and the appearance of senescent-associated distension of satellites and telomere-associated DNA damage foci [32,33]. We aimed to determine the effect of OAC chemotherapy regimens on the induction of a senescent-like phenotype in OAC cells using the well-established marker β-gal^+^ as a marker of senescent-like cells.

**Fig. 5:**
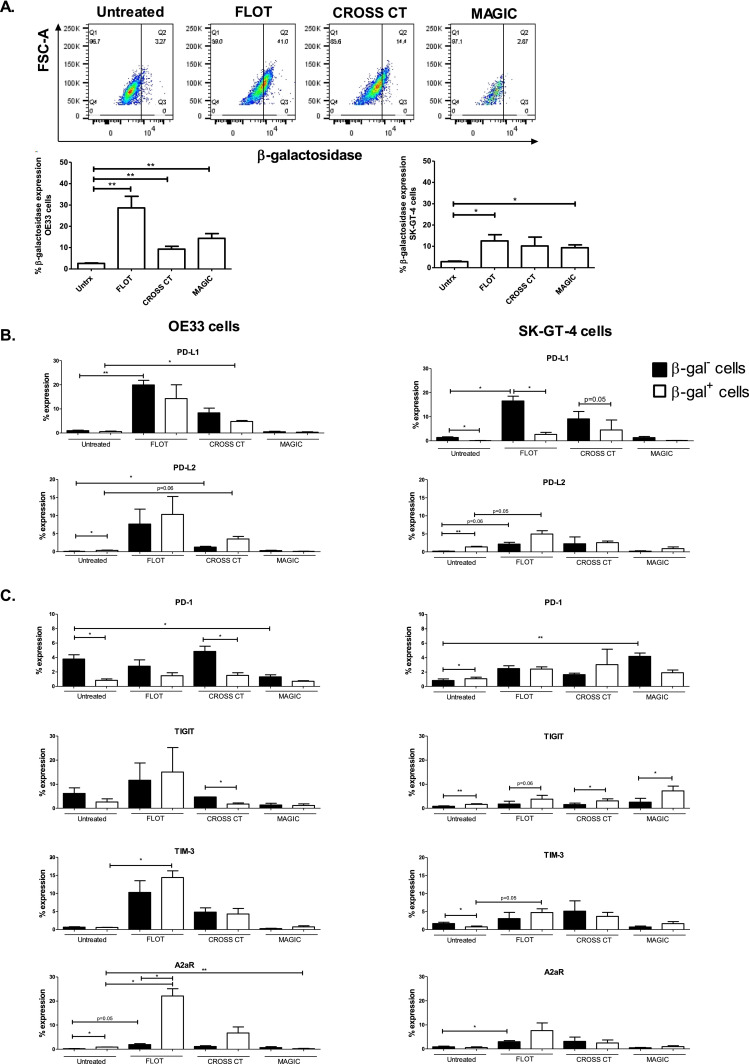
The FLOT chemotherapy regimen enhances a senescent-like phenotype and preferentially upregulates A2aR on the surface of senescent-like OAC cells *in vitro*. OE33 and SK-GT-4 cells were untreated or treated with FLOT, CROSS CT and MAGIC chemotherapy regimens for 48 h *in vitro.* Representative dot plots showing the percentage of viable cells positive for β-galactosidase expression basally (untrx) and following FLOT, CROSS CT and MAGIC chemotherapy regimens in OE33 cells (A). The percentage of viable senescent-like (β-galactosidase positive) OAC cells was determined by flow cytometry (*n* = 3) (A). The percentage of viable senescent (β-gal^+^) and non-senescent (β-gal^−^) OAC cells expressing inhibitory IC ligands (B) and inhibitory IC receptors (C) was determined by flow cytometry (*n* = 3). Paired parametric t-test, *p < 0.05 and **p < 0.01.

Following FLOT, CROSS CT and MAGIC treatment the percentage of OE33 cells expressing the senescent marker β-gal^+^ was significantly increased (untreated: 2.55 ± 0.3% vs. FLOT: 28.59 ± 5.5%, (*p* = 0.005), CROSS CT: 9.28 ± 1.3% (*p* = 0.004) and MAGIC: 14.33 ± 2.3%, (*p* = 0.002)), (Fig. 5A.). Following FLOT and MAGIC treatment the percentage of SK-GT-4 cells expressing β-gal^+^ was also significantly increased (untreated: 2.80 ± 0.4% vs. FLOT: 12.55 ± 2.9%, (*p* = 0.03) and MAGIC: 9.34 ± 1.4% (*p* = 0.01)). However, CROSS CT treatment did not sigificantly alter the percentage of SK-GT-4 cells expressing β-gal^+^ (Fig. 5A.).

Given that FLOT, CROSS CT and MAGIC regimens induced the expression of the senescene marker β-galactosidase in OAC cells *in vitro* we sought to investigate if ICs are expressed on these OAC cells and whether chemotherapy upregulates them further (Fig. 5B.). As ICs play an important role in immune evasion the expression of ICs on senescent OAC cells may represent a drug targetable mechanism of immune evasion. Following FLOT treatment the percentage of senescent OE33 cells expressing TIM-3 was significantly increased (untreated: 0.54 ± 0.1% vs. FLOT: 14.40 ± 1.9% (*p* = 0.02)) (Fig. 5C.). Similarly, following FLOT treatment the percentage of OE33 cells expressing A2aR was significantly increased (untreated: 0.85 ± 0.1% vs. FLOT: 22.10 ± 3.0% (*p* = 0.02)) (Fig. 5C.). There was no significant differences observed in the SK-GT-4 cell line (Fig. 5C.). We also found that TIGIT was expressed at significnatly higher levels on the surface of β-gal^+^ SK-GT-4 compared with β-gal^−^SK-GT-4 cells, basally and following FLOT, CROSS CT and MAGIC treatment (but not OE33 cells). Overall, FLOT had the greatest effect at inducing the senescence marker β-galactosidase in OAC cells and enriched β-gal^+^ OE33 cells for TIM-3 and A2aR expression.

### A subpopulation of OAC cells expressing inhibitory IC ligands and inhibitory IC receptors were identified in OAC tumour tissue biopsies *ex vivo*

Following our *in vitro* studies demonstrating that OAC cells express a range of inhibitory IC ligands and receptors, which are upregulated following chemotherapy treatment on stem-like and senescent-like OAC cells, we screened treatment-naïve (*n* = 10), post-FLOT chemotherapy (*n* = 5) and post-CROSS chemoradiotherapy (*n* = 5) OAC tumour biopsies for the expression of a range of inhibitory IC ligands and receptors *ex vivo*. OAC cells were characterised as CD45^−^CD31^−^ as previously reported [21].

**Fig. 6:**
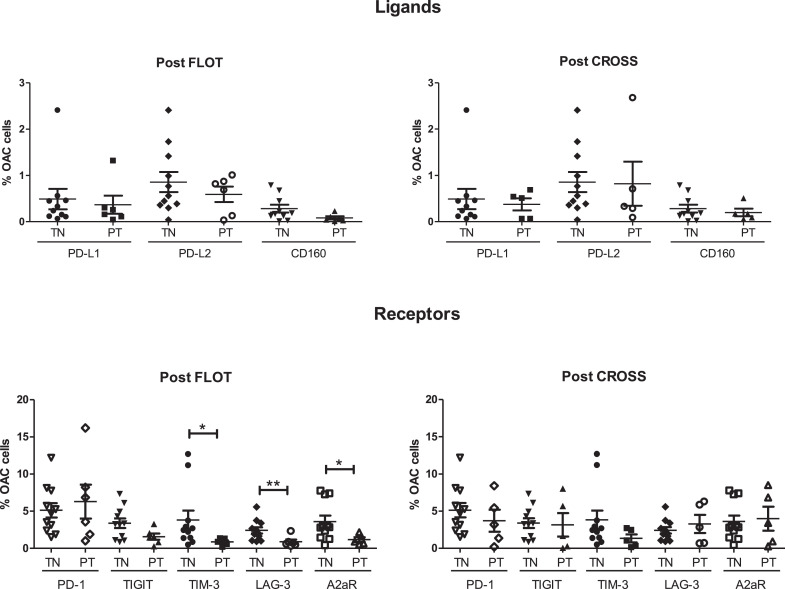
TIM-3, LAG-3 and A2aR IC receptors are expressed at significantly lower levels on viable OAC cells in post-FLOT but not post-CROSS treatment tumour tissue biopsies. OAC cells characterised as CD45^−^CD31^−^ were screened for the surface expression of a panel of inhibitory IC ligands (PD-L1, PD-L2 and CD160) and receptors (PD-1, TIGIT, TIM-3, LAG-3 and A2aR) in OAC tumour tissue biopsies at the treatment-naïve (TN) setting at time of diagnosis (*n* = 10) and post-neoadjuvant treatment (PT) at surgical resection (*n* = 10) *ex vivo* using flow cytometry. Post-treatment: FLOT (*n* = 5) and CROSS (*n* = 5). Mann Whitney test *p < 0.05 and **p < 0.01.

A subpopulation of OAC cells expressing inhibitory IC ligands and receptors were identified in the treatment-naïve, post-FLOT and post-CROSS setting *ex vivo* (Fig. 6.). The percentage of OAC cells expressing PD-L1 in treatment-naïve, post-FLOT and post-CROSS OAC tumour biopsy tissue was: 0.49 ± 0.22%, 0.4 ± 0.2% and 0.37 ± 0.1%, respectively. The percentage of OAC cells expressing PD-L2 in treatment-naïve, post-FLOT and post-CROSS OAC tumour biopsy tissue was: 0.28 ± 0.1%, 0.08 ± 0.03% and 0.20 ± 0.1%, respectively. CD160 was identified on 0.86 ± 0.2%, 0.59 ± 0.2% and 0.82 ± 0.5% of OAC cells in treatment-naïve, post-FLOT and post-CROSS OAC tumour biopsy tissue, respectively (Fig. 6.). PD-1 was identified on 5.13 ± 1.0%, 6.30 ± 2.3% and 3.72 ± 1.5% of OAC cells in treatment-naïve, post-FLOT and post-CROSS tumour biopsy tissue, respectively. The percentage of treatment-naïve, post-FLOT and post-CROSS OAC cells expressing TIGIT was 3.40 ± 0.6%, 1.60 ± 0.4% and 3.15 ± 1.6%, respectively (Fig. 6.). TIM-3 was identified on 3.83 ± 1.2%, 0.88 ± 0.2% and 1.35 ± 0.5% of OAC cells in treatment-naïve, post-FLOT and post-CROSS tumour biopsy tissue, respectively. The percentage of OAC cells expressing LAG-3 in treatment-naïve, post-FLOT and post-CROSS tumour biopsy tissue was: 2.42 ± 0.4, 0.89 ± 0.3% and 3.29 ± 1.2%, respectively (Fig. 6.). A2aR was identified on 3.6 ± 0.8%, 3.62 ± 0.8% and 4.0 ± 1.6% in treatment-naïve, post-FLOT and post-CROSS tumour biopsy tissue, respectively (Fig. 6.). Post-FLOT chemotherapy treatment the percentage of OAC cells expressing TIM-3, LAG-3 and A2aR were significantly lower (*p* = 0.0235, *p* = 0.0077 and *p* = 0.0414, respectively). There was no significant difference between IC expression in the treatment-naïve cohort compared to the post-treatment cohort for the other IC ligands and receptors.

Following identification of a subpopulation of OAC cells expressing inhibitory IC ligands and receptors *in vitro* and *ex vivo,* we investigated the correlative relationship between IC expression on patient OAC tumour cells and clinical patient characteristics and tumour features (Table 1). We observed a strong correlation between IC expression on OAC cells with the expression of additional ICs in the treatment-naïve and post-treatment setting of OAC patients. There was a strong and significant positive correlation (*r* = 0.81) between the percentage of OAC tumour cells expressing LAG-3 and treatment response (*p* = 0.03) in the treatment-naïve setting (Table 1.). In the post-treatment setting there was a strong negative correlation between the percentage of OAC tumour cells expressing PD-L2 (*r*=−0.83), Lag-3 (*r*=−0.70) and A2aR (*r*=−0.72) with treatment response, clinical T stage and serosal invasion, respectively (*p* = 0.002, 0.03 and 0.02, respectively) (Table 1.).

**Table 1:** Correlation of IC expression with IC expression on OAC cells and clinical characteristics and features in the treatment-naïve and post-treatment setting.

Treatment-naïve cohort			
IC protein	Correlated variable	Spearman r	*p*value (two-tailed)
PD-L1	PD-L2	0.67	0.03000
CD160	PD-L2	0.69	0.03000
CD160	LAG-3	0.71	0.02000
CD160	PD-1	0.68	0.03000
PD-L2	CD160	0.69	0.03000
PD-L2	LAG-3	0.70	0.03000
TIGIT	PD-1	0.65	0.04000
LAG-3	CD160	0.71	0.02000
LAG-3	PD-L2	0.70	0.03000
LAG-3	PD-1	0.66	0.04000
A2aR	PD-1	0.66	0.04000
PD-1	CD160	0.68	0.03000
PD-1	TIGIT	0.65	0.04000
PD-1	LAG-3	0.66	0.04000
PD-1	A2aR	0.66	0.04000
CD160	weight	−0.75	0.01000
PD-L2	weight	−0.71	0.02000
PD-1	weight	−0.73	0.04000
LAG-3	treatment response	0.81	0.03000
PD-1	weight	−0.73	0.02000
*Post-treatment cohort*			
*IC protein*	*correlated variable*	*Spearman r*	*p value (two-tailed)*
PD-L1	TIM-3	0.63	0.03885
PD-L1	LAG-3	0.69	0.01857
TIM-3	PD-L1	0.63	0.03885
TIM-3	A2aR	0.87	0.00045
LAG-3	PD-L1	0.69	0.01857
A2aR	TIM-3	0.87	0.00045
PD-L2	treatment response	−0.83	0.00172
LAG-3	clinical T stage	−0.70	0.02529
A2aR	serosal invasion	−0.72	0.01844

Only significant correlations shown. Spearman correlation. Spearman r 0.4–0.59 moderate, 0.6–0.79 strong and 0.8–1 very strong. *p < 0.05.

### Blockade of PD-1 axis intrinsic-signalling in OAC cells reduces the viability of OAC cells inducing apoptosis and enhancing chemotherapy toxicity *in vitro*

Nivolumab and atezolizumab are among the few ICIs approved for clinical use and have demonstrated some success in the clinic, therefore, we investigated whether blocking the PD-1 axis could reduce the viability of OAC cells and induce direct cytotoxicity *in vitro*. In this study we have demonstrated that chemotherapy regimens upregulate PD-1 and PD-L1 on OAC cells. Therefore, we investigated if combining nivolumab, atezolizumab or dual nivolumab-atezolizumab treatment combined with FLOT or CROSS CT regimens could enhance cytotoxicity in OE33 cells and SK-GT-4 cells following 48 h treatment.

**Fig. 7:**
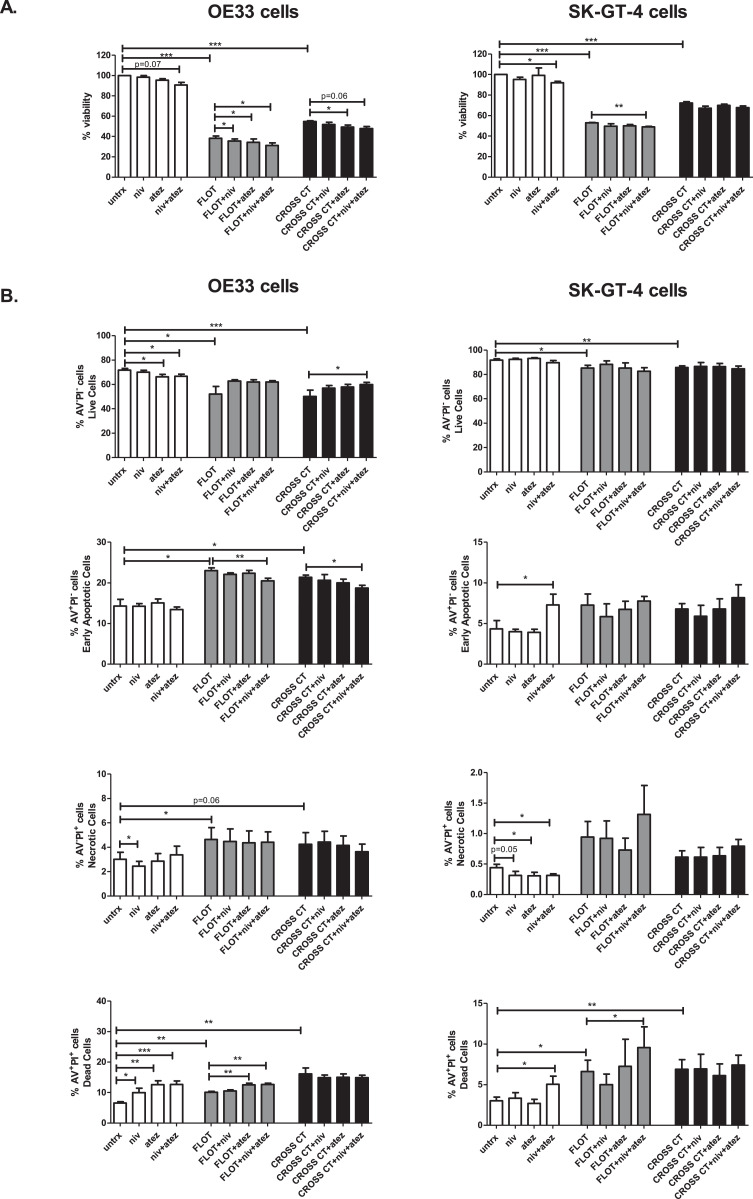
Nivolumab monotherapy and dual nivolumab and atezolizumab enhances the toxicity of FLOT chemotherapy regimen in OAC cells. OE33 cells and SK-GT-4 cells were treated with and without FLOT or CROSS CT regimen in the absence or presence of single agent nivolumab (niv, 10 μg/ml), atezolizumab (atez, 10 μg/ml) or dual nivolumab (10 μg/ml) and atezolizumab (10 μg/ml) for 48 h. Viability was determined by CCK-8 assay and expressed as percentage viability of vehicle control ± SEM (*n* = 4 experimental repeats including *n* = 3 technical replicates) (A). Cell death was also determined by annexin V propidium iodide assay by flow cytometry. Viable cells (AV^−^PI^−^), early stage apoptotic cells (AV^+^PI^−^), necrotic cells (AV^−^PI^+^) and dead cells (AV^+^PI^+^) were assessed (*n* = 5 experimental repeats) (B). *p < 0.05, **p < 0.01, ***p < 0.001, paired parametric *t*-test.

Single agent nivolumab or atezolizumab did not significantly reduce the viability of OE33 cells or SK-GT-4 cells (Fig. 7A.). Although, a trend toward a decrease in viability in OE33 cells following 48 h treatment with dual nivolumab-atezolizumab was observed. However, dual nivolumab-atezolizumab significantly reduced the viability of SK-GT-4 cells (100% vs. 91.89 ± 1.5%, respectively (*p* = 0.01)) (Fig. 7A.). Interestingly, single agent nivolumab significantly enhanced the toxicity of FLOT demonstrated by a significant reduction in viability compared to the FLOT only control in OE33 cells (FLOT: 38.32 ± 2.2% vs. FLOT + nivolumab: 35.56 ± 2.0% (*p* = 0.03)), (Fig. 7A.). Similar trends were observed for single agent atezolizumab in OE33 cells (FLOT: 38.32 ± 2.2% vs. FLOT + atezolizumab: 34.29 ± 3.3% (*p* = 0.04)), (Fig. 7A.). Additionally, dual nivolumab-atezolizumab significantly enhanced the toxicity of FLOT demonstrated by a significant reduction in the viability of OE33 cells compared to the FLOT only control (FLOT: 38.32 ± 2.2% vs. FLOT + nivolumab-atezolizumab: 31.27 ± 2.4%, (*p* = 0.01)). Similar trends were observed in SK-GT-4 cells (Fig. 7A.). Single agent atezolizumab significantly enhanced the toxicity of CROSS CT demonstrated by a significant reduction in viability compared to the CROSS CT only control in OE33 cells (CROSS CT: 54.64 ± 0.9% vs. CROSS CT + atezolizumab: 49.25 ± 2.1% (*p* = 0.03)), (Fig. 7A.). There was a trend toward an enhancement in CROSS CT toxicity using dual nivolumab-atezolizumab treatment demonstrated by a significant reduction in viability compared to the CROSS CT only control in OE33 cells (CROSS CT: 54.64 ± 0.9% vs. CROSS CT + nivolumab-atezolizumab: 47.82 ± 2.0% (*p* = 0.06)), (Fig. 7A.). Single agent or dual checkpoint blockade did not significantly enhance CROSS CT toxicity in SK-GT-4 cells by CCK-8 assay (Fig. 7A.).

Single agent nivolumab, atezolizumab and dual nivolumab-atezolizumab significantly increased the percentage of dead OE33 cells (defined as double positive Annexin V and PI cells) (untreated: 6.57 ± 0.5% vs. nivolumab: 9.99 ± 1.4% (*p* = 0.03), atezolizumab: 12.63 ± 1.2% (*p* = 0.003), and nivolumab-atezolizumab: 12.68 ± 1.1% (*p* = 0.0002)) (Fig. 7B.). Similarly, dual nivolumab-atezolizumab significantly increased the percentage of dead SK-GT-4 cells (untreated: 3.± 0.5% vs. nivolumab-atezolizumab: 5.03 ± 1.0% (*p* = 0.02)) (Fig. 7B.).We also found that single agent atezolizumab and dual nivolumab-atezolizumab enhanced the toxicity of FLOT in OE33 cells demonstrated by a significant increase in the percentage of dead cells compared to the FLOT treated control (FLOT: 10.07 ± 0.4% vs. FLOT + atezolizumab: 12.55 ± 0.5% (*p* = 0.002) and FLOT + nivolumab-atezolizumab: 12.64 ± 0.4% (*p* = 0.001)) (Fig. 7B.). Similarly, dual nivolumab-atezolizumab in combination with FLOT significantly increased the percentage of dead SK-GT-4 cells compared to the FLOT only control (FLOT: 6.61 ± 1.4% vs. FLOT + nivolumab-atezolizumab: 9.57 ± 2.6% (*p* = 0.03)) (Fig. 7B.).

Overall, we found that blockade of the PD-1 axis significantly reduced OAC cell viability and enhanced apoptosis induction, particularly enhancing the toxicity of the FLOT chemotherapy regimen *in vitro.*

**Fig. 8:**
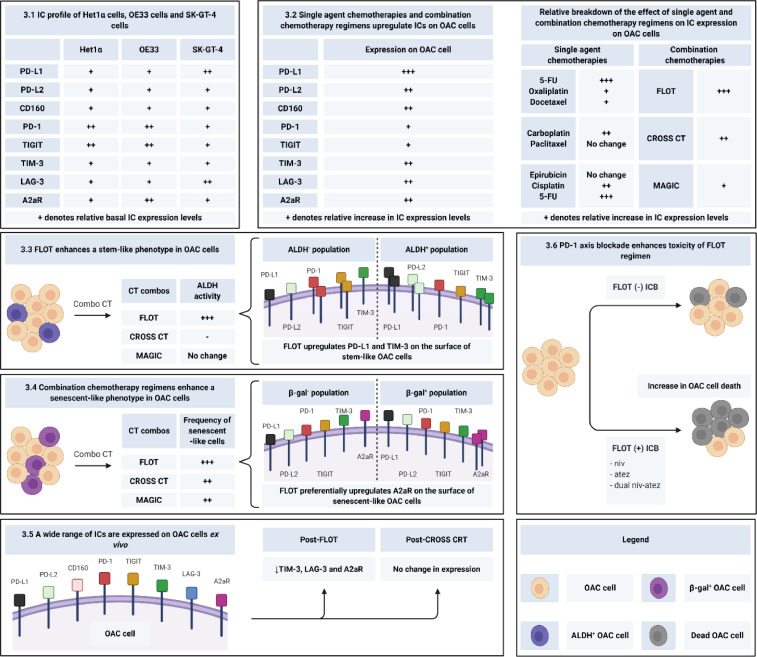
Graphical summary of the key findings from the study. Each section of the figure corresponds to the key findings of this study. Section 3.1 displays the relative expression levels of ICs on Het1A, OE33 and SK-GT-4 cells. Section 3.2 summarises the relative effect of single agent chemotherapies and combination chemotherapy regimens on IC expression. A table showcasing the breakdown of the relative effect of single agent chemotherapies and their combinations on IC expression is also included. Section 3.3 highlights the effect of chemotherapy regimens on a stem-like phenotype in OAC cells and highlights that FLOT preferentially upregulates PD-L1 and TIM-3 on the surface of stem-like OAC cells *in vitro*. Section 3.4 demonstrates that combination chemotherapy enhance a senescent-like phenotype in OE33 cells and that FLOT preferentially upregulates A2aR on the surface of OAC cells *in vitro*. Section 3.5 highlights the expression levels of ICs on OAC cells in tumour tissue and demonstrates that post-FLOT TIM-3, LAG-3 and A2aR IC receptors are significantly decreased on OAC cells (characterised as CD45^−^CD31^−^) *ex vivo*. Section 3.6 shows that treatment with nivolumab, atezolizumab or dual nivolumab-atezolizumab enhances the toxicity of the FLOT regimen *in vitro*.

The key findings from our study are summarised in Fig 8.

## Discussion

We demonstrated that the FLOT chemotherapy regimen enhanced a more stem-like and senescent-like phenotype in OAC cells and perhaps may be selecting for a more therapeutically resistant phenotype. Interestingly, the CROSS CT regimen significantly reduced ALDH activity in OE33 cells. This may suggest that the chemotherapies comprising the CROSS CT regimen may be attenuating a stem-like phentoype in certain OACs. Our study demonstrates that FLOT, CROSS CT and MAGIC chemotherapy regimens enhance a senescent-like phenotype in OAC cells *in vitro,* whereby again we saw that FLOT substantially enhanced a senescent-like phenotype. Chemotherapy regimens are cytotoxic to tumour cells and often promote immunogenic cell death, this study highlights the double-edged sword of chemotherapy, where we observed that the FLOT regimen in particular may also elicit tumour promoting effects through selecting for a more aggressive cancer cell phenotype [10].

Traditionally, it was widely accepted that inhibitory IC ligands and receptors were exclusively expressed on cancer cells and immune cells, respectively. However, emerging studies have shown that inhibitory IC receptors PD-1 [13], [14], [15], [16], TIM-3 [17,18], TIGIT [19] and A2aR [20] are also expressed on lung, melanoma, colorectal [19], cervical [17] and gastric [18,20] cancer cells. Our study identified a subpopulation of OAC cells expressing inhibitory IC receptors PD-1, TIGIT, TIM-3, LAG-3, A2aR and inhibitory IC ligands PD-L1, PD-L2 *in vitro* and *ex vivo*. A complementary study by Kollmann D. et al., showed that PD-1 was expressed on 77% of OAC patient tumour cells (*n* = 168) at levels greater than 5%, determined by immunohistochemical analysis and was associated with significantly decreased 5-year overall survival compared to those who lacked PD-1-expressing OAC cells in their tumours (43.5% *versus* 68.8%, respectively) [34]. Further work is needed to determine the functional relevance of specific IC receptor expression by OAC tmour cells.

The percentage of OAC cells in tumour tissue expressing LAG-3, A2aR and TIM-3 were significantly lower post-FLOT treatment compared with the treatment-naïve setting. However, treatment of OE33 and SK-GT-4 cells with FLOT for 48 h *in vitro* significantly increased IC expression on the surface of OAC cells including LAG-3, A2aR and TIM-3. The *in vitro* experiments recapitulate the direct effects of chemotherapy on IC expression on OAC cells using an *in vitro* culture system. In contrast, the analysis of IC expression in tumour biopsy tissue encapsulates the effect of the entire tumour microenvironment on IC expression in the treatment-naïve setting as well as the combined direct and indirect effects of FLOT and CROSS CRT ~6 weeks post-treatment. It is also likely that changes in IC expression dynamically and longitudinally occur over this time.

Interestingly, we observed that single agent chemotherapies upregulate IC ligands and receptors on the surface of OAC cells, whereby 5-FU had the most substantial effect. Additionally, the FLOT regimen consistently upregulated both inhibitory IC ligands and receptors on OAC cells *in vitro* to a greater extent than the CROSS CT and MAGIC chemotherapy regimens. ICs play an integral role in maintaining immunotolerance and are key players in mediating tumour immune evasion, therefore this suggests that FLOT may induce immunogenic cell death in OE33 and SK-GT-4 cells *in vitro*. Studies have shown that oxaliplatin and 5-FU, which comprise the FLOT regimen have immunostimulatory properties and induce immunogenic cell death in lung and colon cancer cells [35,36]. Complementary studies have aslo shown that 5-FU increases PD-L1 on the surface of OE33 and HCT-116 cells [28] and similarly, cisplatin increases PD-L1 on the surface of lung cancer cells [27].

FLOT and CROSS CT treatments had the greatest effect in upregulating PD-L1, PD-L2, CD160, TIM-3, LAG-3 and A2aR ICs on OAC cells *in vitro.* However, PD-1 and TIGIT were only minimally increased. Further studies are required to further determine how biologically significant this is as PD-1 and TIGIT are expressed on only a sub-population of OAC cells *in vitro* and *in vivo* and therefore, they may be expressed on aggressive cancer cell clones that often exist in low frequencies and PD-1 and TIGIT signalling pathways may contribute to their survival or treatment resistance.

The upregulation of ICs on the surface of OAC cells suggests these ICs may offer some level of protection against chemotherapy or perhaps they may be upregulated in an attempt to repair the chemotherapy-induced damage to the cell. Emerging studies have shown that IC receptors and ligands directly enhance glycolysis [38], proliferation [13], invasion, migration [23,39] and DNA repair [40] via cancer cell-intrinsic signalling. Several studies have demonstrated that DNA damage signalling upregulates PD-L1 expression on the surface of cancer cells [41], [42], [43] and PD-L1 cancer cell-intrinsic signalling mediates DNA repair [42,43]. Blockade of PD-L1 on the surface of OAC cells could potentially prevent repair of the chemotherapy-induced DNA damage thereby, enhancing chemotherapy toxicity. It is unclear whether the viable OAC cells that survived chemotherapy treatment have upregulated inhibitory ICs on their cell surface or if the chemotherapy treatment selectively kills OAC cells that lack inhibitory IC expression enriching for OAC cells that express inhibitory ICs. The former may suggest that OAC cells upregulate inhibitory ICs perhaps as a survival advantage to facilitate immune evasion or perhaps IC-intrinsic signalling is promoting immune-independent mechanisms of resistance to chemotherapy-induced cell death. The latter suggests that ICs are expressed on the surface of OAC cells that are more resistant to chemotherapy-induced cell death and persist following treatment. Our results demonstrate that chemotherapy preferentially upregulates PD-L1 and TIM-3 ICs on a more stem-like OAC cell phenotype *in vitro*. Additionally, FLOT chemotherapy significantly upregulated TIM-3 and A2aR on a subpopulation of senescent-like OAC cells. Other studies have shown that PD-L1 is enriched on cancer stem cells and provides a mechanism of immune escape [12]. TIM-3 has also been identified on cervical and gastric cancer cells and induced invasion and migration of HeLa cells *in vitro* [17]. High levels of TIM-3 expression on gastric cancer cells correlated with metastasis in gastric cancer patients [18]. Shi et al., demonstrated that A2aR signalling via PI3K-AKT-mTOR upregulated stemness-associated and EMT-like proteins in gastric cancer cells *in vitro* and that A2aR knockout murine models resulted in a decrease in the number and size of micrometastatic lesions in the lungs of mice [20]. Ultimately, the expression of inhibitory IC ligands and receptors on OAC cells may function in tandem to offer OAC cells a survival advantage via promoting a range of cancer hallmarks.

Our study demonstrates for the first time that single agent and combination ICIs reduce OAC cell viability and induce apoptosis in OAC cells directly and independent of the immune system, a novel finding in the context of OAC. Further studies are required to determine if blockade of PD-1 or PD-L1 could reduce tumour cell growth and induce tumour cell death via immune-independent mechanisms using *in vivo* pre-clinical models which will help further elucidate the biological role and clinical potential for targeting these pathways to reduce tumour growth in OAC.

Furthermore, we investigated the potential for combining ICIs with chemotherapy to enhance chemotherapy-induced OAC cell death. Although we did not observe a substantial amount of synergism between ICI-chemotherapy combinations we demonstrated that combining ICIs with chemotherapy had a limited but significant effect in enhancing chemotherapy-induced OAC cell death. Similarly, Liu. et al., demonstrated that PD-1 blockade enhanced chemosensitivity to 5-FU in a 5-FU resistant gastric cancer cell line [37]. These important clinically relevant findings do question whether ICIs might synergise with combination chemotherapy regimens in patients to enhance chemotherapy-induced OAC cell death in an immune-independent manner and highlights the need for further studies to answer this question. In addition, these findings may be reflective of the limited benefit observed from clinical trials testing combination ICI-chemotherapy regimens, but this may translate to a measurable improvement in clinical outcomes. We also demonstrated that PD-L1 is upregulated on stem-like OAC cells *in vitro* and therefore, blockade of the PD-1 signalling axis may be reducing the survival of stem-like OAC cells and subsequently enhancing the efficacy of chemotherapy which could translate to a clinically meaningful improvement in outcomes for patients. This may account for the limited effect observed between the addition of ICIs to chemotherapy as stem-like cells only comprise a subpopulation of a tumour cell population.

## Future perspective

A therapeutic rationale is highlighted for combining ICIs with the FLOT, CROSS and MAGIC regimens in OAC to prevent immune evasion mediated through chemotherapy-induced upregulation of ICs on OAC cells. The expression of inhibitory IC ligands or receptors on the surface of stem-like and senescent-like OAC cells may represent a mechanism of immune escape for these OAC cell clones. IC blockade may therefore facilitate immune-mediated clearance of stem-like and senescent-like OAC cells in patients and enhance clinical outcomes. Further studies are required to determine if the stem-like and senescent-like OAC cells are truly cancer stem cells and senescent OAC cells, respectively. Importantly, future studies are warranted to determine the biological and clinical significance of IC-intrinsic signalling in either promoting the survival or function of both tumour cells and the inherently treatment resistant cancer stem cells.

## Specific author contributions

All authors have contributed to this manuscript. Maria Davern collected and processed samples, carried out experiments, performed data analysis, interpreted the results and contributed to conception and design of the study. Noel E. Donlon and Andrew Sheppard collected patient samples and carried out experiments. Fiona O’ Connell performed data analysis. John V. Reynolds, Narayanasamy Ravi, Dermot O'Toole and Anshul Bhardwaj were involved in collecting and processing clinical samples and data. Stephen G. Maher and Niamh Lynam-Lennon contributed to design of the project. Joanne Lysaght made substantial contribution to the conception and design of the project, interpretation of the data, securing funding and overall supervision of the project. All authors were involved in the drafting and critical appraisal of the manuscript.

## Funding

This work was funded by an Irish Research Council scholarship (GOIPG/2017/1659) and the CROSS oesophageal cancer research charity.

## Declaration of Competing Interest

The authors declare that they have no known competing financial interests or personal relationships that could have appeared to influence the work reported in this paper.

## Appendix Supplementary materials

Image, application 1
