# Supplementary material for: Chemotherapy regimens induce inhibitory immune checkpoint protein expression on stem-like and senescent-like oesophageal adenocarcinoma cells
Source: Transl Oncol. 2021 Mar 22;14(6):101062. doi: 10.1016/j.tranon.2021.101062 (PMC8008239; doi:10.1016/j.tranon.2021.101062)
Supplement: Supplementary file 1 [file mmc1.docx]

18^th^ February 2021

Dear Günter Schneider,

Ms. Ref. No.:  TRANON-D-21-00046

The authors would like to thank the editor and reviewers for their helpful comments and time taken to review this manuscript. We have addressed the editor’s and reviewers’ comments in order below.

Editor’s comments:

The study is relevant and addresses an important clinical aspect. There are two major concerns, seen also by the former reviewers of Cancer Letters, needed to be addressed to generate a translational advance essential for publication:

1. The Editor agrees with the reviewers that the study is descriptive and that mechanistic insights are needed. There are numerous ways to elaborate such insights.

Thank you for your comments, we have now elaborated on potential mechanistic insights in the discussion section, please see below for your convenience:

*“Interestingly, we observed that single agent chemotherapies upregulate IC ligands and receptors on the surface of OAC cells, whereby 5-FU had the most substantial effect. Additionally, the FLOT regimen consistently upregulated both inhibitory IC ligands and receptors on OAC cells in vitro to a greater extent than the CROSS CT and MAGIC chemotherapy regimens. ICs play an integral role in maintaining immunotolerance and are key players in mediating tumour immune evasion, therefore this suggests that FLOT may induce immunogenic cell death in OE33 and SK-GT-4 cells in vitro. Studies have shown that oxaliplatin and 5-FU, which comprise the FLOT regimen have immunostimulatory properties and induce immunogenic cell death in lung and colon cancer cells^38,39^. Complementary studies have aslo shown that 5-FU increases PD-L1 on the surface of OE33 and HCT-116 cells^28^ and similarly, cisplatin increases PD-L1 on the surface of lung cancer cells^27^.*

*The upregulation of ICs on the surface of OAC cells suggests these ICs may offer some level of protection against chemotherapy or perhaps they may be upregulated in an attempt to repair the chemotherapy-induced damage to the cell. Emerging studies have shown that IC receptors and ligands directly enhance glycolysis^42^, proliferation^13^, invasion, migration^23,43^ and DNA repair^44^ via cancer cell-intrinsic signalling. Several studies have demonstrated that DNA damage signalling upregulates PD-L1 expression on the surface of cancer cells^45,46,47^ and PD-*

*L1 cancer cell-intrinsic signalling mediates DNA repair^46,47^. Blockade of PD-L1 on the surface of OAC cells could potentially prevent repair of the chemotherapy-induced DNA damage thereby, enhancing chemotherapy toxicity. It is unclear whether the viable OAC cells that survived chemotherapy treatment have upregulated inhibitory ICs on their cell surface or if the chemotherapy treatment selectively kills OAC cells that lack inhibitory IC expression enriching for OAC cells that express inhibitory ICs. The former may suggest that OAC cells upregulate inhibitory ICs perhaps as a survival advantage to facilitate immune evasion or perhaps IC-intrinsic signalling is promoting immune-independent mechanisms of resistance to chemotherapy-induced cell death. The latter suggests that ICs are expressed on the surface of OAC cells that are more resistant to chemotherapy-induced cell death and persist following treatment. Our results demonstrate that chemotherapy preferentially upregulates PD-L1 and TIM-3 ICs on a more stem-like OAC cell phenotype in vitro. Additionally, FLOT chemotherapy significantly upregulated TIM-3 and A2aR on a subpopulation of senescent-like OAC cells. Other studies have shown that PD-L1 is enriched on cancer stem cells and provides a mechanism of immune escape^12^. TIM-3 has also been identified on cervical and gastric cancer cells and induced invasion and migration of HeLa cells in vitro^17^. High levels of TIM-3 expression on gastric cancer cells correlated with metastasis in gastric cancer patients^18^. Shi Z. et al., demonstrated that A2aR signalling via PI3K-AKT-mTOR upregulated stemness-associated and EMT-like proteins in gastric cancer cells in vitro and that A2aR knockout murine models resulted in a decrease in the number and size of micrometastatic lesions in the lungs of mice^20^. Ultimately, the expression of inhibitory IC ligands and receptors on OAC cells may function in tandem to offer OAC cells a survival advantage via promoting a range of cancer hallmarks.”*

1. There are concerns about the relevance of the synergism between the chemotherapy/ICI inhibitor combination. E.g. the effects in the viability assays are very small (Fig. 7), which holds also for the FACS analysis. Therefore, a more robust evaluation of the combination therapy is needed to demonstrate the clinical potential.

Thank you for these insights, we have now addressed your concerns within our discussion highlighting the limited synergy observed in our assays between combination chemotherapy and ICIs however, we clearly underline the important translational potential of these findings in OAC that warrants publication. Please see below for your convenience:

*“Our study demonstrates for the first time that single agent and combination ICIs reduce OAC cell viability and induce apoptosis in OAC cells directly and independent of the immune system, a novel finding in the context of OAC. Further studies are required to determine if blockade of PD-1 or PD-L1 could reduce tumour cell growth and induce tumour cell death via immune-independent mechanisms using in vivo pre-clinical models which will help further elucidate the biological role and clinical potential for targeting these pathways to reduce tumour growth in OAC.*

*Furthermore, we investigated the potential for combining ICIs with chemotherapy to enhance chemotherapy-induced OAC cell death. Although we did not observe a substantial amount of synergism between ICI-chemotherapy combinations we demonstrated that combining ICIs with chemotherapy had a limited but significant effect in enhancing chemotherapy-induced OAC cell death. Similarly, Liu, N. et al., demonstrated that PD-1 blockade enhanced chemosensitivity to 5-FU in a 5-FU resistant gastric cancer cell line^41^.* *These important clinically relevant findings do question whether ICIs might synergise with combination chemotherapy regimens in patients to enhance chemotherapy-induced OAC cell death in an immune-independent manner and highlights the need for further studies to answer this question. In addition, these findings may be reflective of the limited benefit observed from clinical trials testing combination ICI-chemotherapy regimens, but this may translate to a measurable improvement in clinical outcomes. We also demonstrated that PD-L1 is upregulated on stem-like OAC cells in vitro and therefore, blockade of the PD-1 signalling axis may be reducing the survival of stem-like OAC cells and subsequently enhancing the efficacy of chemotherapy which could translate to a clinically meaningful improvement in outcomes for patients. This may account for the limited effect observed between the addition of ICIs to chemotherapy as stem-like cells only comprise a subpopulation of a tumour cell population.”*

Reviewer #2:

Davern et al., investigates immune checkpoint receptor and ligand expression in oesophageal adenocarcinoma (OAC) cell lines in response to different treatment regimens. They observe differences in expression in cells with stem- and senescent-like phenotype. The authors addressed most of the comments brought up by the original reviewers.

1. However, lack of reproducibility of the observations in human tissue remains a major concern.

Thank you for your comment. We would like to highlight that the post-treatment tumour biopsies are sampled at the time of surgical removal of the tumour, which is 6 weeks post-chemotherapy or chemoradiotherapy treatment. Therefore, it is not an accurate comparison to directly compare the IC profile of OAC cells from tumour biopsies 6-weeks post-treatment with OAC cell lines treated with chemotherapy for 48h in vitro. Tumour cells are highly adaptive and plastic, therefore, when the chemotherapy is no longer present in the TME we would expect to see the IC profile change or perhaps return to the baseline expression prior to treatment. The value of the in vitro studies is to examine the direct effect of chemotherapy on the epithelial cells themselves. We address this in the discussion, please see below for your convenience:

*“**The percentage of OAC cells in tumour tissue expressing LAG-3, A2aR and TIM-3 were significantly lower post-FLOT treatment compared with the treatment-naïve setting. However, treatment of OE33 and SK-GT-4 cells with FLOT for 48h in vitro significantly increased IC expression on the surface of OAC cells including LAG-3, A2aR and TIM-3. The in vitro experiments recapitulate the direct effects of chemotherapy on IC expression on OAC cells using an in vitro culture system. In contrast, the analysis of IC expression in tumour biopsy tissue encapsulates the effect of the entire tumour microenvironment on IC expression in the treatment-naïve setting as well as the combined direct and indirect effects of FLOT and CROSS CRT 6 weeks post-treatment. It is also likely that changes in IC expression dynamically and longitudinally occur over this 6 week time period”*

1. Based on the gates provided in Supplementary Figure 1, the specificity of the staining is not clear. The authors can use CD45+ fraction to demonstrate true positivity.

Thank you for this comment. Our study profiles the IC expression profiles on epithelial OAC cells *ex vivo*, therefore we use CD45^-^CD31^-^ double negativity to confirm that these are cancer cells and not immune cells as previously carried out in^1^.

1. Figure 4. Based on the scatter, there is a significant concern with the dot plots that ALDH positive cells are simply dead. The authors state that a viability dye was included but please show the representative plots. Also please include representative plots from unstained/treated samples as background control to establish true ALDH positivity. Due to the differences in scatter background signal for the checkpoint inhibitors could be different. Therefore, it is important to correct for these baseline artifact/differences.

Thank you for your comment. The IC expression profile and ALDH activity was only profiled on live cells using the zombie viability dye to exclude dead cells from the analysis. Please see below the gating strategy in Figure S4. that we have now included to address your comment. The DEAB negative ((-)ve) control was used to identify the gating strategy for IC co-expression with ALDH^-^ and ALDH^+^ OAC cells. This accounted for the differences in scatter background signal and the baseline fluorescence of OAC cells to ensure that accurate gating was used to characterise IC expression on ALDH^-^ and ALDH^+^ OAC cells.


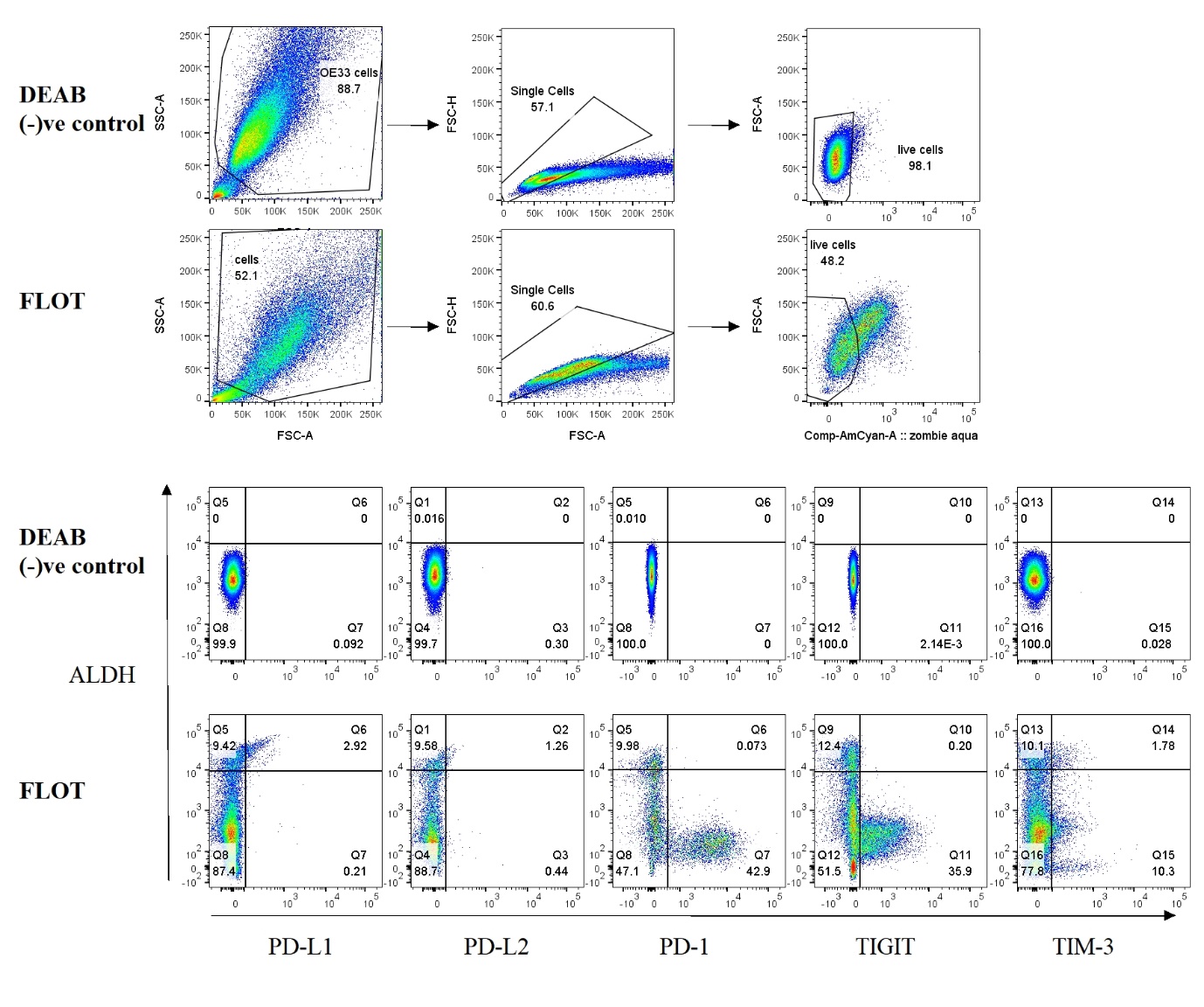


**Fig. S4. Gating strategy for assessing co-expression of IC ligands and receptors on the surface of ALDH^-^ and ALDH^+^ OAC cell lines *in vitro*.** Gate 1 included all cells in the FSC versus SSCA plot, doublet cells were then excluded using FSC-H versus FSC-A plot, dead cells were excluded using zombie aqua viability marker. The surface co-expression of inhibitory IC ligands and receptors on ALDH^-^ and ALDH^+^ OAC cells was then assessed on this live, doublet excluded population. Representative dot plots are shown for the DEAB negative ((-)ve) control and stained FLOT OE33 cells for the expression of PD-L1, PD-L2, PD-1, TIGIT and TIM-3 on the surface of ALDH^-^ and ALDH^+^ OE33 cells *in vitro*.

1. There are significant differences in the level of IC receptor expression between Figure 3 and 4. Can the authors please discuss this discrepancy?

Thank you for your observation. The expression of ICs is different in figure 3 and 4 as figure 4 includes antibody panels that contain the aldefluor dye However, the key objective of figure 4 is to examine the effect of chemotherapy on IC expression on ALDH^-^ and ALDH^+^ OAC cells and therefore the changes in IC expression on ALDH^-^ and ALDH^+^ OAC cells is relative compared to the control and can provide a reliable depiction of the changes in the IC expression profile following chemotherapy treatment.

Minor point:

1. Please consider changing "food-pipe" with esophagus, which is better use of terminology

*Thank you, this has now been corrected.*

1. For supplementary Figure 2, standard dose-response curves would be a better way of demonstrating the data.

Thank you, we have now included standard dose-response curves instead. Please see below for your convenience.

**Fig. S2. Clinically-relevant single agent chemotherapies display increasing cytotoxicity in a dose-dependent manner against OAC cells in *vitro* following 48h**. OE33 cells and SK-GT-4 cells were treated with a range of increasing concentrations of single agent 5-FU, oxaliplatin (oxali), docetaxel (doc), epirubicin (epi), cisplatin (cis), carboplatin (cbx) and paclitaxel (pac) for 48h. Cell viability was determined by CCK-8 assay (n=3). Paired parametric t test, *p<0.05, **p<0.01, ***p<0.001.

1. Percentages and SDs in the text make it difficult to follow.

Thank you for this observation, we understand this, however, the journal specifies that they would like percentages and SDs. We provide a summary of the key findings at the end of each section and a graphical summary of the important data to help the reader follow the study.

1. It is important to discriminate between biological and statistical significance. In Figure 3, PD1 and TIGIT expression increase appears to be extremely limited.

*Thank you, we have highlighted this in the discussion, please see below for your convenience:*

*“FLOT and CROSS CT treatments had the greatest effect in upregulating PD-L1, PD-L2, CD160, TIM-3, LAG-3 and A2aR ICs on OAC cells in vitro. However, PD-1 and TIGIT were only minimally increased. Further studies are required to further determine how biologically significant this is as PD-1 and TIGIT are expressed on only a sub-population of OAC cells in vitro and in vivo and therefore, they may be expressed on aggressive cancer cell clones that often exist in low frequencies and PD-1 and TIGIT signalling pathways may contribute to their survival or treatment resistance.”*

1. Y-axis in Figure 3 and 4 should be arranged to better depict the data.

Thank you, this has now been altered to ensure that the numbering on the y-axis is the same for both OE33 and SK-GT-4 cell lines. However, some of the ICs are upregulated more on one cell line compared to the other and therefore, to ensure the reader can easily observe and compare this difference we think it is more appropriate to maintain the same axis on both graphs.

On behalf of myself and my co-authors, I would once again like to thank the Editor and the reviewers for their helpful and insightful comments.

Yours sincerely,


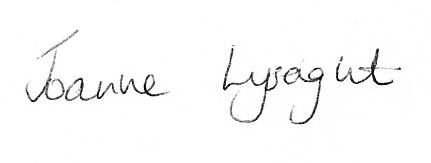


Joanne Lysaght, Ph.D.
